# Supplementary material for: Compensating cortical thickness for cortical folding-related variation
Source: Imaging Neurosci (Camb). 2026 Jan 23;4:IMAG.a.1106. doi: 10.1162/IMAG.a.1106 (PMC12836397; doi:10.1162/IMAG.a.1106)
Supplement: Supplementary Material [file IMAG.a.1106_supp.pdf]

Supplementary Materials

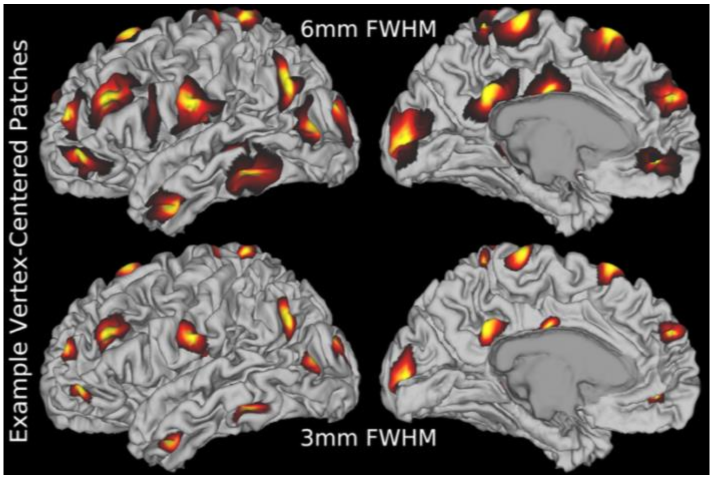

Supplementary Figure 1: Two example vertex-centered patches within the selected search range for optimal patch size (3–10 mm). The minimum size of 3 mm is sufficient to include at least one inward and one outward fold, which is necessary for capturing local folding geometry relevant to our analysis. Medial wall vertices are masked and excluded from all our analyses. Data are available at http://balsa.wustl.edu/66vrZ.

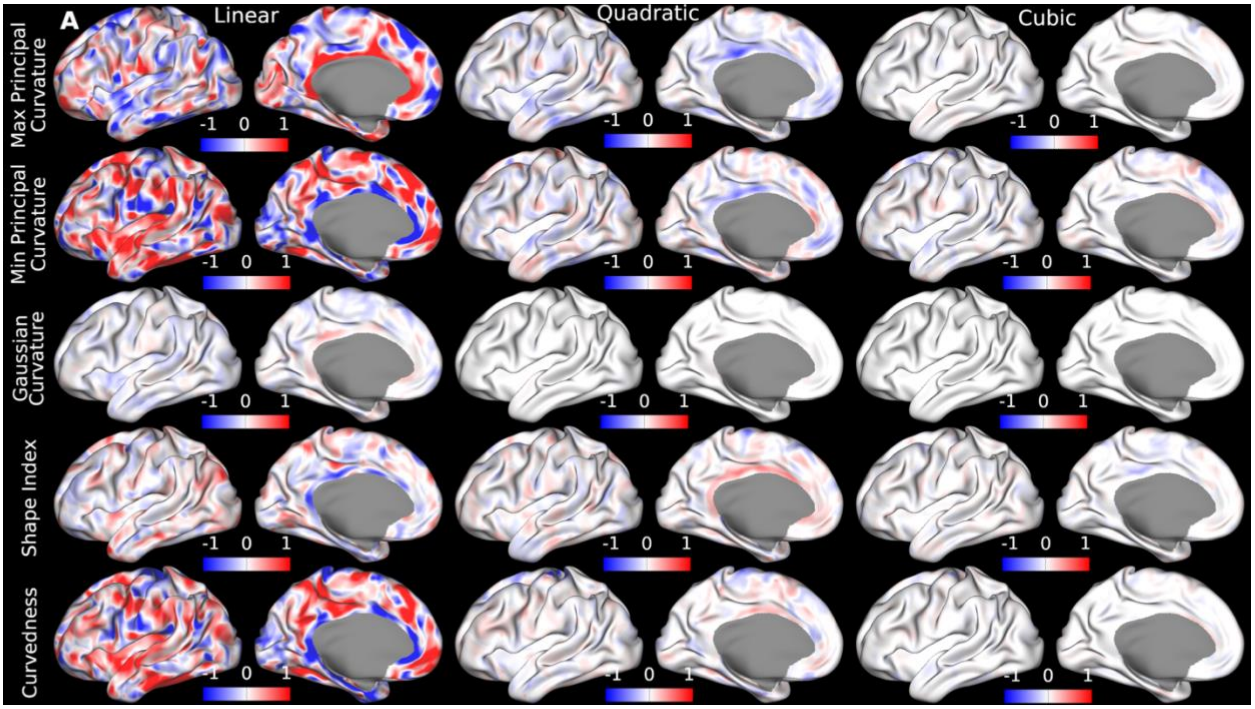

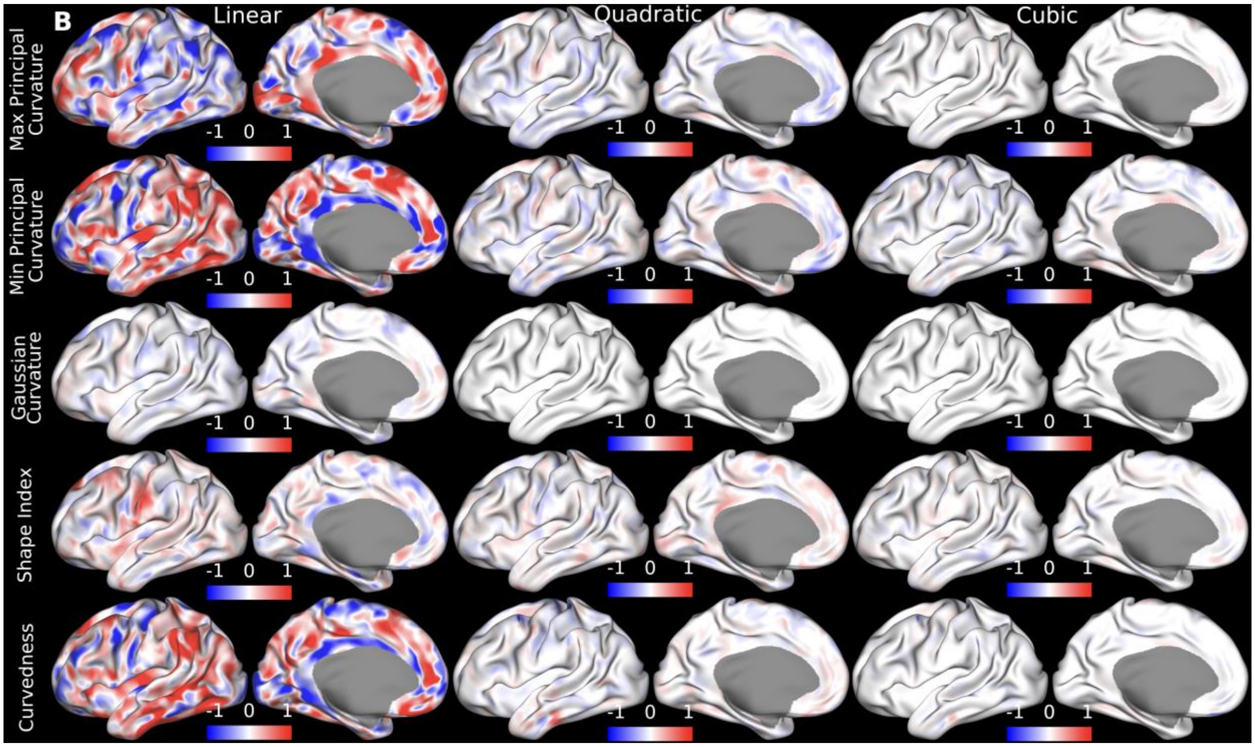

Supplementary Figure 2: Vertex-wise (normalized with the standard deviation of the corresponding curvature feature) regression coefficient maps displayed on the inflated surface of two individuals of the HCP-YA dataset (A: case 100408, B: case 100307). Red and blue represent positive and negative modeled effect of a curvature feature on thickness, respectively. Cortical thickness is more strongly associated with principal curvatures and curvedness and less strongly with Gaussian curvature, as Gaussian curvature fails to disambiguate between a cup and a cap morphology. Linear regression coefficients show the strongest effects, while quadratic terms contribute moderately and cubic terms minimally. Accordingly, we included terms up to the second order in our polynomial model, as cubic coefficients account for only negligible effects. Data are available at http://balsa.wustl.edu/1r95D and http://balsa.wustl.edu/56rXD.

Supplementary Table 1: Summary of full width at half-maximum (FWHM) values used for cortical thickness smoothing in prior studies. This table lists representative smoothing kernel sizes reported in the literature, illustrating the range of values commonly applied in surface-based cortical thickness analyses. In our review, if the smoothing parameter is presented in σ (standard deviation) we converted it into FWHM for consistency.

**Resource**	**FWHM (mm)**
(Chung et al., 2005)	30
(Doyle-Thomas et al., 2013)	20
(Hurtz et al., 2014)	10
(Hutton et al., 2008)	12
(Kim et al., 2012)	10
(Kuperberg et al., 2003)	60
(Lerch et al., 2005)	20
(Misaki et al., 2012)	15

(Nam et al., 2015)	20
(Rosas et al., 2002)	16
(Salat et al., 2004)	52
(Lerch and Evans, 2005)	30
